# Exploring the feasibility and acceptability of couple-based psychosexual support following prostate cancer surgery: study protocol for a pilot randomised controlled trial

**DOI:** 10.1186/1745-6215-15-183

**Published:** 2014-05-24

**Authors:** Jane M Robertson, Gerard J Molloy, Prasad R Bollina, Daniel M Kelly, S Alan McNeill, Liz Forbat

**Affiliations:** 1 Cancer Care Research Centre, School of Nursing, Midwifery and Health, University of Stirling, Stirling FK9 4LA, UK; 2 School of Psychology, National University of Ireland, University Road, Galway, Ireland; 3 Urology Department, Western General Hospital, Crewe Road South, Edinburgh EH4 2XU, UK; 4 School of Nursing and Midwifery Studies, Cardiff University, Eastgate House, Newport Road, Cardiff CF24 0AB, UK

**Keywords:** Adjustment, Communication, Counselling, Couple, Functioning, Intervention, Prostate cancer, Psychological, Quality of life, Relationship, Sexual

## Abstract

**Background:**

Men who undergo surgery for prostate cancer frequently experience significant side-effects including urinary and sexual dysfunction. These difficulties can lead to anxiety, depression and reduced quality of life. Many partners also experience psychological distress. An additional impact can be on the couple relationship, with changes to intimacy, and unmet psychosexual supportive needs in relation to sexual recovery and rehabilitation. The aim of this exploratory randomised controlled trial pilot study is to determine the feasibility and acceptability of a novel family-relational-psychosexual intervention to support intimacy and reduce distress among couples following prostate cancer surgery and to estimate the efficacy of this intervention.

**Methods/Design:**

The intervention will comprise six sessions of psychosexual and relationship support delivered by experienced couple-support practitioners. Specialist training in delivering the intervention will be provided to practitioners and they will be guided by a detailed treatment manual based on systemic principles. Sixty-eight couples will be randomised to receive either the intervention or standard care (comprising usual follow-up hospital appointments). A pre-test, post-test design will be used to test the feasibility of the intervention (baseline, end of intervention and six-month follow-up) and its acceptability to couples and healthcare professionals (qualitative interviews). Both individual and relational outcome measures will assess sexual functioning, anxiety and depression, couple relationship, use of health services and erectile dysfunction medication/technologies. An economic analysis will estimate population costs of the intervention, compared to usual care, using simple modelling to evaluate the affordability of the intervention.

**Discussion:**

Given the increasing incidence and survival of post-operative men with prostate cancer, it is timely and appropriate to determine the feasibility of a definitive trial through a pilot randomised controlled trial of a family-relational-psychosexual intervention for couples. The study will provide evidence about the components of a couple-based intervention, its acceptability to patients and healthcare professionals, and its influence on sexual and relational functioning. Data from this study will be used to calculate sample sizes required for any definitive trial.

**Trial registration:**

ClinicalTrials.gov Identifier: NCT01842438.

Registration date: 24 April 2013; Randomisation of first patient: 13 May 2013

## Background

Surgery is a major treatment modality for prostate cancer [1] and has been hypothesised to reduce mortality more than other treatments for localised prostate cancer [2]. However, the risks of surgery are known to include erectile dysfunction [3] even when nerve-sparing approaches are used [4]. Long-lasting sexual and urinary difficulties are the most common and troubling side-effects following radical prostatectomy [5] alongside loss of libido, ejaculatory dysfunction, orgasmic dysfunction and penile shortening [6]. Even at 24 months post-operatively, most men have not returned to their baseline measure of sexual function prior to surgery [7]. These side-effects can be distressing for men and their partners due to the impact on psychological well-being and couple intimacy [8-11].

Many men affected by prostate cancer and erectile dysfunction experience symptoms of anxiety and depression [12,13], with reduced quality of life directly associated with urinary and sexual body-image changes that occur after surgery [14]. Partners of these men also experience considerable psychological distress [9,15-17], with anxiety and depression often reported at higher levels for partners than for patients [18,19]. The difficulties experienced after surgery can also impact on the couple relationship itself; for instance, erectile dysfunction is associated with reduced wellbeing [20,21] and lower levels of dyadic adjustment in the first year after surgery [22].

Prostate cancer has been described as a ‘relationship disease’ [23] and it has been established that couples affected by prostate cancer often have significant unmet psychosexual supportive care needs around sexual recovery and rehabilitation [24,25]. With such evidence of the impact of sexual dysfunction on relationships, there has been increasing interest in the development of couple-based psychosocial interventions that aim to improve wellbeing and relationship quality. Couple-based interventions have been effective in reducing psychological distress [26] and facilitating healthy communication [27,28]. Sexual rehabilitation therapy has been particularly helpful in increasing use of erectile dysfunction medications [29] and re-establishing a sexual relationship [30,31].

Evidence therefore exists to support the efficacy of couple-based interventions for reducing psychological distress, enhancing couple communication and improving relationship functioning among couples affected by cancer [32]. Interventions for couples affected by prostate cancer have targeted sexual functioning [31], relationship functioning [33], or combined both aspects [19,28,29,34,35]. Reviewed systematically, the overall effectiveness of such interventions remains inconclusive [36], although manualised face-to-face interventions that address the connection between sexual difficulties and relationship variables have shown improvements in sexual functioning, including erectile function. Chisholm *et al*. [36] conclude that the evidence remains weak due to methodological limitations, such as small sample sizes and ineffective outcome measures, while longer-term gains in functioning have been limited.

Of particular note, interventions have not been successful in improving both relationship and sexual functioning [36]. Consequently, it would be beneficial to devise an intervention framework that has a lasting impact on sexual *and* emotional aspects of the couple relationship. Studies suggest that understanding family-of-origin relationships may be important in supporting sexual functioning in marriage [37], and that to address sexual issues it is critical to support the relationship more generally [38]. Consequently, an approach which is family-relational and psychosexual combines key elements of a potentially fruitful intervention.

The current study offers a family-relational-psychosexual approach to supporting the couple, by combining family systems principles with elements of sex therapy. Combining these approaches enables the intervention to address broader relational issues that impact specific problems around sex and intimacy. The intervention therefore has the potential for long-term benefits to participants, as the wider context of prostate cancer and couple dynamics are a focus. It will develop a new way of supporting couples that combines family systems principles with elements of sex therapy to enhance intimacy and reduce distress. A family systems approach is based on an understanding that the family/relational context influences couple dynamics, which consequently impact on relational and psychosexual outcomes. The treatment model adopts a comprehensive approach to couple support, addressing communication and relationships within the family context, to address broader relational issues in which specific problems around sex and intimacy will be located.

### Aims

The primary aim of the study is to determine whether a family-relational-psychosexual intervention is feasible and acceptable for couples affected by prostate cancer in the context of one care-delivery setting. Subsidiary to this, the study aims to understand what processes of randomisation are plausible for a larger trial, determine a sample-size calculation for a definitive trial, and estimate the efficacy of the intervention to increase sexual and relationship functioning.

### Objectives

1. Design a family-relational-psychosexual treatment for delivering a couple-based intervention.

2. Determine the acceptability and feasibility of the intervention to patients, partners and healthcare professionals in the context of one care-delivery setting.

3. Determine sample size, recruitment and randomisation processes for a definitive trial.

4. Establish long-term cost implications to be addressed in a definitive trial.

## Methods

### Design

This study has been designed to develop and assess the feasibility of a couple-based intervention following prostate cancer surgery. The design encompasses phases i/ii of the Medical Research Council (MRC) complex intervention framework [39,40]. It embeds a two-arm pilot randomised controlled trial (RCT) followed by qualitative interviews assessing acceptability and feasibility of the intervention.

A pre-test, post-test design is being used to test the feasibility of the intervention (baseline, end of intervention and six-month follow-up). The study is underpinned by Realistic Evaluation methodology, which acknowledges the complexity of interventions and the contexts in which they are delivered and assessed [41]. This methodology ensures that data is gathered from a range of relevant stakeholders to identify facilitators and barriers to delivery and uptake. The aim is also to produce findings that will be of relevance in routine clinical practice.

### Setting and participants

Patients with prostate cancer and their partners are being recruited from a single site in one National Health Service (NHS) board: an outpatient surgical urology clinic at the Western General Hospital, Edinburgh (NHS Lothian), UK. The recruiting clinic sees patients from a wide catchment area covering four Health Boards in Scotland: the Borders; Dumfries and Galloway; Fife; the Lothians. Recruitment will take place over one year.

#### Eligibility

Eligible patients are all men who: (1) are >11 weeks post-operative for localised prostate cancer (to recruit men who have recovered from the immediate effects of surgery and who have consequently started to regain some functioning); (2) have received surgery in the previous two years (since long-term adaptation will have commenced in patients who have had surgery beyond this timeframe); (3) have a partner (in an established same-sex or different-sex relationship); (4) score ≤60 (the clinical threshold for potency [42]) on the sexual function domain of the Expanded Prostate Cancer Index Composite (EPIC).

Patients are excluded from the study if: (1) they have a prognosis of ≤1 year (most men who have had recent surgery will have a good prognosis, so it is unlikely that many men will be excluded by this criteria); (2) they reside in Dumfries and Galloway (to prevent excess burden travelling to the intervention site); (3) they cannot provide informed consent; (4) they are unable to communicate in English (this is a pilot trial, and if we move to a full-scale trial in future we would seek to include interpreters/translators).

#### Sample size calculation

The trial aims to recruit 68 men and partners at routine follow-up after surgery, with 30% over-recruitment to enable adjustment for loss to follow-up [43]. A sample size of 68 couples was calculated using G-Power [44] based on having 90% power to detect a small-to-medium effect size *f* of 0.20 on the primary outcome (using repeated measures analysis of variance (ANOVA) and testing the within-between group interaction in this two-group design with two points of measurement (end of intervention and six-month follow-up) at an alpha level of 0.05). A small-to-medium effect on sexual function was selected based on previous intervention effects seen for couple interventions in the cancer literature [45].

Assuming a baseline mean of 33 and a standard deviation of 24 on the sexual function subscale of the EPIC [46], this study will have 90% power to detect a mean difference of 9.6 units at the 0.05 level. This will allow the study to estimate the efficacy of this intervention on the mean difference in overall sexual function (as measured by EPIC) between the intervention and control groups pre- and post-test. The standardised difference observed will be used to calculate the sample size required for a definitive trial to test the efficacy of this intervention. The anticipated effect size of *f* of 0.20 on the primary outcome is a crude estimate, as it is based on a pooled estimate of a heterogeneous selection of couple interventions for several types of cancer [45].

Based on surgical data from the locale (2009 to 2011) we estimate that there will be 500 patients who will be between 12 weeks and two years from surgery during the one-year recruitment period. The estimated recruitment rate from a prior study with a similar population and intervention design was 21% [33]. As some of the patient population will be excluded from the study based on the eligibility criteria above, we anticipate contacting approximately 420 patients to recruit 88 couples (taking account of 30% over-recruitment [43] to achieve a sample size of 68 couples completing the study).

### Phase 1: designing the intervention

The intervention will be informed by the extant literature regarding the pragmatics and focus for the couple work. It will consist of six sessions [34,35] conducted every two to three weeks [28,47] by registered practitioners at a local voluntary sector counselling organisation. The intervention will be delivered off-site in central Edinburgh, away from the pressures of a busy outpatient hospital clinic where there is usually limited time to discuss psychosexual concerns during healthcare consultations [24].

The content of the intervention will comprise assistance with emotional disclosure [33,48], psycho-education [19,29], relational and sexual needs [29-31] and dyadic adjustment and coping [49,50]. A treatment manual will be developed to guide delivery of the intervention [51]. The manual will comprise information about prostate cancer and its effects, principles of therapeutic change, guidance on using the manual and a detailed session structure plan. This manual-based family-relational-psychosexual support will be based on systemic principles [52-55] combined with techniques from sex therapy, that is, sensate focus [56]. Therefore, the manual aims to integrate components of systemic theory with elements of sex therapy to support intimacy and emotional aspects of the couple relationship.

Specialist training in delivery of the intervention will be provided to practitioners holding accredited counselling or psychotherapy qualifications. Training will include sessions led by men affected by prostate cancer and a systemic practitioner in cancer care. Fidelity to the manual will be assessed as an ongoing process, recorded by practitioner self-report using a checklist provided by the research team.

In line with routine counselling interventions, this study adopts a waiting-list control design [57]. The control group will be offered the intervention if the analysis indicates a significant benefit in quality of life for couples who receive the support.

The session structure for the intervention will cover six main topic areas as follows:

**Session 1 - Getting to know the couple: orientation and engagement.** This first session will outline the support on offer. Topics for discussion will include:

● the couple’s definition of current issues, concerns and problems

● the cancer diagnosis and treatment(s)

● the partner’s role in the context of diagnosis and treatment

● mapping the support network and wider family system

**Session 2 - The couple’s communication style and relationship.** This session is primarily focused on understanding the patient and their partner as a couple, to explore how they convey love, support, understanding, companionship and affection.

**Session 3- Intergenerational patterns of illness, coping and affection.** Focusing on intergenerational patterns, discussion will centre on the role and meaning of illness in the couple relationship in the context of:

● family resilience

● dyadic adjustment in ill-health

● the role of partners and family when someone is ill

● how people in the family express intimacy

**Session 4 - Couple intimacy before and after cancer.** Intimacy before and after cancer will be explored. A psycho-educational approach will be used to promote closeness and express intimacy after treatment. The place of medical treatments will be considered and techniques from sex therapy will be applied if appropriate for the couple.

**Session 5 - Further exploration of emerging areas.** This session will focus on areas that have emerged in previous sessions where the practitioner and couple wish to give more time to them. This may include more work on increasing levels of intimacy, and improving satisfaction with sexual activity, with discussion of successes or challenges therein.

**Session 6 - Summarising the couple’s accomplishments and future planning.** The final session will summarise work to date, with discussion of relapse prevention and how to take forward progress that has been made, including the setting of short- and long-term goals. This will include a specific focus on maintaining intimacy and dyadic adjustment.

### Phase 2: determining the parameters for a definitive trial

#### Patient recruitment

Eligible patients will be identified from the urology surgical lists at the Western General Hospital, Edinburgh, UK. The research nurse at the site will apply eligibility criteria before patients are invited to complete the EPIC questionnaire. Patients will be invited to complete the questionnaire via two routes: either on-site at their post-operative follow-up appointment at the outpatient surgical urology follow-up clinic; or by postal invitation from the clinical team.

The recruiting site will complete logs to report recruitment rates at baseline and any attrition following randomisation. Additionally, attendance at the couple support sessions will be logged to assess attrition rates at each stage of the intervention. The recruitment rate for a larger definitive trial will be calculated from the time taken for 68 couples to complete the study.

##### Route 1: outpatient surgical urology follow-up clinic

Participants will be recruited from the outpatient surgical urology follow-up clinic. Clinic nurses will approach potentially eligible patients and invite them to complete the EPIC screening measure. Participants who are eligible based on the EPIC score will be verbally notified, given detailed information about the study, and provided with baseline questionnaires and consent forms. A member of the research team will be on hand to answer any questions that patients may have.

When a nurse is not available, the study will be introduced by the consultant/registrar during the consultation; if interested in the study, the patient will then be introduced to the researcher, who will provide detailed information about the study and invite them to complete EPIC.

##### Route 2: postal invitation from clinical team

This route will be adopted on days when the clinic is very busy and there is consequently limited private opportunity for completing the EPIC screening tool. A letter and detailed information sheet is sent to patients who are potentially eligible. The letter invites them to complete and return the EPIC questionnaire. The research team will write to tell patients whether or not they are eligible; if eligible, the remaining baseline questionnaires and consent forms are also issued at this time.

#### Randomisation, allocation concealment and blinding

Once consent is gained and baseline data (outcome measures and demographics) are returned, patients will be randomly assigned using block randomisation with a 1:1 allocation ratio to receive the intervention or standard care comprising their usual follow-up hospital appointments. The allocation sequence is generated using computer software (GraphPad Software, La Jolla, CA, USA) that randomly assigns subjects to the control and intervention arms in blocks of 20. This block randomisation process ensures equal numbers in the intervention and control arms and removes selection bias [58].

A research administrator, who has no involvement in the study, will assign participants to the control or intervention arm according to the allocation sequence described above. This individual will not be aware of the allocation sequence until the moment of assignment.

In line with other designs of this type [59], blinding will not be possible due to the nature of the intervention and the acceptability component of the study. It will be clear to study participants that they have been allocated to the intervention or control group due to the timing of data collection relative to receipt of the intervention.

In order to establish if the intervention and control group are clinically similar, the two groups will be compared by physical health status. Consequently, the hospital’s audit officer will provide the research team with anonymous data relating to clinical markers of disease pathology. The markers are: (1) prostate-specific antigen (PSA), a protein produced by the prostate that can be measured in a man’s blood serum and used as a prognostic indicator; when combined with (2) the Gleason score (GS), to differentiate the grade of tumour (from low- to high-grade malignancy); along with (3) the tumour, node and metastases (TNM) staging system to distinguish localised from locally advanced and metastatic disease (the degree of spread and involvement of lymph nodes) [60]. This clinical data will allow the team to determine whether physical health status may be a confounding variable in explaining any variance in the results of the trial.

In addition, ineligibility data will be recorded noting number of patients and the reasons for their ineligibility. Reasons for non-participation or withdrawal from the study will be recorded to understand the acceptability or otherwise of the support. However, in keeping with ethical practice, potential participants will not be asked for reasons for non-participation; this will only be noted if proactively provided by patients or partners.

**Figure 1:**
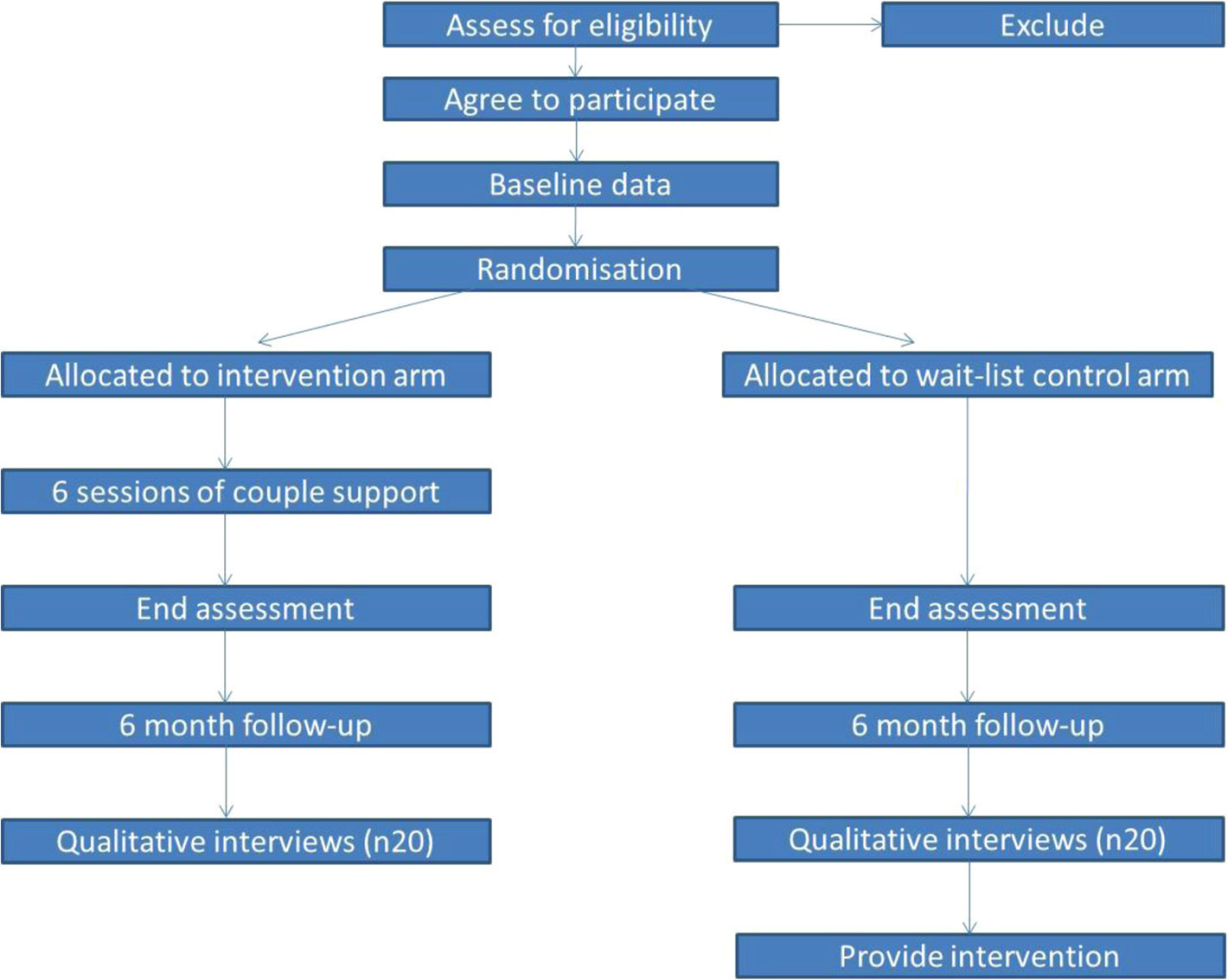
CONSORT schema.

The recruitment and randomisation process is set out in the CONSORT schema in Figure 1.

#### Data collection

As this is a feasibility study, it is not formally testing hypotheses. Nevertheless, we will examine outcome measures pre/post/follow-up (baseline, end of intervention and six-month follow-up) to evaluate the indicative effect of the intervention on both individual and couple/relational issues. Outcome measures have been selected to prevent over-burdening participants (by capping the length and number of measures used). We have generously estimated that all measures may take 30 minutes to complete, but feel that many participants will complete them in 10 minutes. Validated measures, detailed below, will be used alongside a specially designed *pro forma* for collating additional information on health-service use and demographic data.

In order to assess the feasibility and acceptability of the intervention, practitioners will complete adherence checklists, indicating for each couple which components of the intervention were covered in the therapy.

#### Outcome measures

The primary outcome measure (see below) assesses sexual functioning as part of a broader health-related quality-of-life measure. The secondary measures reflect the evidence that men and their partners require psychological (anxiety, depression), relational and sexual components to an intervention to meet the needs of this group.

Primary outcome measure

● Sexual functioning - EPIC measures general and sex-specific items related to prostate cancer [46]. EPIC is a comprehensive instrument that evaluates patient function and ‘bother’ after prostate cancer treatment. Potency of ≤60 in the sexual function domain is a clinical cut-off [42].

Secondary outcome measures

● Anxiety and depression - Hospital Anxiety and Depression score (HADS) measures psychological impact [61].

● Functional couple relationship - systemic core outcome measure (SCORE 15) measures relationship changes [62].

● Use of health services and erectile dysfunction medication/technologies, collated via a self-report questionnaire designed by the investigators.

Outcome measures will be assessed at baseline, at end of intervention and six months later.

#### Statistical analysis

Although hypothesis testing should proceed cautiously in a feasibility/acceptability study, we anticipate that couples in the intervention group will show increased scores for sexual functioning (measured using EPIC) and functional couple relationship (SCORE 15 measure), and decreased scores for anxiety and depression (HADS measure).

The study will therefore estimate the efficacy of this intervention based on the mean difference in primary (EPIC) and secondary outcome measures (SCORE 15, HADS) between the intervention and control groups pre- and post-test. Baseline demographic characteristics will be reported as mean and standard deviation for continuous data and number (percentage) for categorical data. ANOVA will be used to test the within-between group interaction in this two-group design, with two points of measurement (end of intervention and six-month follow-up). Data will be managed and analysed using SPSS version 19 (IBM Corp, Armonk, NY, USA). The significance level applied to all analyses (and any adjustments for *post hoc* analyses) will be set at 0.05.

Analysis of the practitioner adherence checklist data will proceed using frequency counts to identify those areas addressed most frequently. This data will be interpreted with reference to the qualitative appraisal of feasibility and acceptability.

### Phase 3: qualitative assessment of acceptability and feasibility

In addition to the calculation of recruitment and retention rates, a qualitative approach will be used in the post-trial evaluation to assess the acceptability of the intervention. Interviews will be digitally recorded, transcribed and managed using NVivo (version 10) software (QSR International, Doncaster, Australia). The analysis of transcripts will begin with thematic analysis, and subsequently draw on systemic theory, to provide an in-depth understanding of the acceptability of the intervention. This is critical in gaining experiential accounts of psychosexual and relational needs at follow-up to evidence subjective outcomes.

#### Patient and partner interviews

Individual face-to-face interviews will be conducted with participants of the intervention group (N = 10 patients and their partners) and control group (N = 10 patients and their partners). Sampling will be purposive, with interviewees stratified for degree of change, to determine qualitative appraisals of either usual care or the intervention and its success or otherwise. Separate interviews will be used to enable the exploration of private accounts of the experience. Participants will be invited to opt in to an interview when completing their post-intervention measures. The timing reflects the desire to gain feedback on the intervention when recall is likely to be strongest.

Participants in the intervention group will be asked about what was most and least helpful about the couple support, the location and timing of this support, their relationship and use of services, and whether anything could be changed or added to the intervention. Participants in the control group will be asked about their routine care, any support they received in relation to their quality of life, sexual functioning, relationship and use of services.

#### Healthcare professional interviews

Post-intervention interviews will be conducted with key-informant healthcare professionals (N = 10), including those who have had experience of delivering the intervention. Interviews will elicit views on the acceptability and feasibility of the content and process of delivery of the intervention; for example, eligibility criteria, method and timing of recruitment, session structure, timing and duration of intervention. The suitability of the intervention within the context of everyday care packages and current prostate cancer provision in this locale will be assessed, to consider questions such as whether the intervention works better because it is off-site and away from clinical pressures. Determining the acceptability and feasibility of this single-site study will act as a starting point from which the parameters of a definitive trial can be determined.

### Establishing long-term cost implications

Economic analysis will focus on additional costs, compared to usual care, and potential cost savings, using simple modelling to evaluate the affordability of the intervention [63]. Data will be collected from the organisation providing the intervention to provide estimates on (i) duration of appointments, (ii) ‘did not attend’ rates, and (iii) additional administrative activities. Data will also be collected from participants on (i) contact with general practitioner (GP), (ii) contact with hospital, (iii) use of additional couple support to estimate cost savings to the NHS. Modelling of this data will be applied to estimate likely volumes and patterns of demand, and staff burden, to estimate population-cost implications. Population costs will be estimated using epidemiological data on prevalence of men post-surgery for prostate cancer, alongside data on attrition from the intervention.

### Ethical considerations

The study has been approved by NHS West of Scotland Research Ethics Committee (Reference: 12/WS/0255). Management approval for recruiting at the Western General Hospital site has been obtained from NHS Lothian. A detailed information sheet will be provided to participants with study information and contact details. Informed consent will be obtained from all patients and partners who agree to participate in the study. Additional consent will be sought from those couples and healthcare professionals who agree to take part in post-intervention acceptability/feasibility interviews. If consent is given by participants, GPs will be advised of their patient’s involvement in the trial. Couples in the trial will consent to having their baseline summary scores communicated to the practitioners delivering the intervention; the use of outcome measures is routine practice and helps practitioners tailor their approach to couples.

#### Data storage and confidentiality

Participants will be assigned a study number. All documents will be labelled with the study number alone, and data stored on computers will only use this number. All names referred to in the interviews will be changed following transcription and saved under the assigned study number. Pre-anonymised transcripts will then be deleted.

Identifiable personal data will only be retained in the following circumstances: consent forms will be stored in a locked filing cabinet; couple names/addresses will be stored in a locked filing cabinet and a password-protected computer used in disseminating outcome measures and final report/s.

#### Risks to participants

People diagnosed and treated for cancer constitute a group that can be considered vulnerable. Consequently, considerable care is required to ensure that adequate safeguards are put in place.

Men recruited to the trial will not be in the immediate aftermath of diagnosis. Recruitment to the study will only occur when the man is at least 11 weeks past surgery. Consequently, with current waiting lists, he is likely to be approached about the study four to five months after diagnosis, and will have recovered considerably from surgery. Further, only men who score ≤60 on the sexual function domain of EPIC (the clinical threshold for potency) will be invited to participate, so that the intervention is stratified to target those men most in need of additional support.

Due to the nature of the disease, and our focus on relational and psychosexual issues, it is likely that interviews touch on sensitive areas. The team has experience of conducting interviews with couples on a range of sensitive issues and is aware of the potential emotional demands on participants. Any participant who feels they are unable to continue with an interview will be reassured that it is permitted to withdraw from the research at any time.

### User involvement

The project has been designed to have user involvement throughout. A group of men affected by prostate cancer have helped design the methods and will continue to act as consultants to the project. They have helped design the intervention, train the practitioners, construct appropriate prompts for focus groups/interview schedules, and will contribute to analysis and dissemination.

## Discussion

Prostate cancer is the most common cancer in men. Surgery is a major treatment modality for localised prostate cancer. However, after surgery, men can suffer from several side-effects like erectile dysfunction, anxiety and decreased quality of life, which impact on wellbeing, intimacy and relationship quality. Men’s partners are also influenced by these side-effects and can often experience distress and reduced wellbeing. Yet previous research has revealed that couples are not generally well supported to cope with these side-effects of surgery.

With an increase in the incidence and survival of postoperative men with prostate cancer, there are significant unmet psychosexual needs for couples affected by prostate cancer. We aim to develop a family-relational-psychosexual intervention for couples and then to test the feasibility of this support in a pilot randomised control trial. We expect that sexual and relationship functioning will be increased in couples who receive the intervention, while distress will be reduced, compared to those couples who receive standard care.

At the end of this study, we will have determined whether the intervention is effective or not, and if it is acceptable to couples and healthcare professionals. An economic analysis will estimate population costs. Therefore, the study will provide evidence about the nature of a couple-based intervention, its acceptability and influence on sexual and emotional aspects of the relationship.

## Trial status

Open - currently recruiting participants.

## Abbreviations

ANOVA: repeated measures analysis of variance; EPIC: Expanded Prostate Cancer Index Composite; GP: general practitioner; GS: Gleason score; HADS: Hospital Anxiety and Depression score; MRC: Medical Research Council (United Kingdom); NHS: National Health Service (United Kingdom); PSA: prostate-specific antigen; RCT: randomised controlled trial; SCORE 15: systemic core outcome measure; TNM: tumour, node and metastases; UK: United Kingdom.

## Competing interests

The authors declare that they have no competing interests.

## Authors’ contributions

JR contributed to the data collection and analysis, manuscript writing and final approval of the manuscript. GM contributed to the data analysis, manuscript writing and final approval of the manuscript. PB contributed to the conception and design, critical revision and final approval of the manuscript. DK contributed to the conception and design, critical revision and final approval of the manuscript. AM contributed to the design, critical revision and final approval of the manuscript. LF contributed to the conception and design, data collection and analysis, manuscript writing and final approval of the manuscript. All authors read and approved the final manuscript.

## Authors’ information

JR is a Researcher in the Cancer Care Research Centre at the University of Stirling. Her research interests centre on quality of life, ageing, caring, cancer and dementia. GM is a Lecturer in the School of Psychology at the National University of Ireland, Galway. His research focuses on the influence of personality and the social environment on health and illness. PB is a Consultant Urological Surgeon at the Western General Hospital, NHS Lothian. He is chair of the South East Scotland Cancer Network. DK is Royal College of Nursing Chair in Nursing Research and Director of Research and Innovation at Cardiff University School of Health Care Sciences. His research focuses on the impact and experience of illness including cancer and palliative care. AM is a Consultant Urological Surgeon at the Western General Hospital, NHS Lothian and Honorary Professor in the School of Engineering and Physical Sciences at Heriot-Watt University. LF is a Reader and Co-Director of the Cancer Care Research Centre at the University of Stirling. She conducts research focusing on families, children and relationships.
